# Predictors of Mortality among Patients Enrolled on Antiretroviral Therapy in Aksum Hospital, Northern Ethiopia: A Retrospective Cohort Study

**DOI:** 10.1371/journal.pone.0087392

**Published:** 2014-01-31

**Authors:** Kidane Tadesse, Fisaha Haile, Neway Hiruy

**Affiliations:** Mekelle University, College of Health Science, Department of Public Health, Mekelle, Ethiopia; Fundacion Huesped, Argentina

## Abstract

**Background:**

Since launching of antiretroviral (ART) treatment, the numbers of patients enrolled in to ART are increasing in many developing countries. But many studies done across Africa including Ethiopia on antiretroviral therapy programs have shown higher mortality at the first six months of treatment initiation. But the factors associated with this high mortality are poorly characterized. So this study aims to determine mortality and identify predictors of it among patients on ART.

**Methods:**

Retrospective cohort study was employed among a total of 520 records of patients who were enrolled on antiretroviral therapy in Aksum hospital from September 2006 to August 2011. Baseline patient records were extracted from electronic and paper based medical records database and analysed using Kaplan Meier survival and Cox proportional hazard model to identify the independent predictors of mortality of patients on ART.

**Results:**

A total of 46 (8.85%) deaths was observed giving an overall mortality rate of 3.2 per 100 person-years. The independent predictor of mortality identified for this cohort were haemoglobin level <11 mg/dl (Hazard Ratio (HR) = 1.9, 95%-CI = 1.01, 3.52), CD4 cell counts lower than 50 cells/µl (HR = 2.1, 95%- CI = 1.13,3.89), Male gender (HR = 1.9, 95%-CI = 1.01,3.52), Weight <40 kg (HR = 2.3,95% CI = 1.24,4.55), primary level of education and lower (HR = 2.6, 95%- CI = 1.29,5.55).

**Conclusions:**

The over all mortality of adults patients on ART was low but higher in the early months of ART initiation. low levels of haemoglobin <11 gm/dl, lower CD4 cell count, male gender, weight <40 Kg and individuals who have primary level of education and lower were indentified as the independent predictors of mortality. For this reason, early initiation of ART despite the CD4 count and method of HIV diagnosis, nutritional support and close monitoring of patients in the early periods of ART treatment initiation is very crucial to improve patient survival.

## Introduction

According to studies done in low and middle income countries, there is countless impact of ART treatment on survival rates of patients. Immune and viral responses were good and mortality reductions were similar in high- and low-income settings under optimal uptake of ART treatment, care and support. But early mortality was higher in low-income settings due to the occurrence of larger proportion of severe opportunistic infections and advanced stage of disease at ART initiation [1], [2]. But, until the end of December 2010, more than 6.6 million people are receiving HIV treatment from 14.2 million eligible people living with HIV in low and middle income countries [1].

How ever, According to a study from America, death rates in a cohort of Adults on ART declined significantly after the introduction of ART [3]. In the western world, the importance of ART in reducing mortality and prevent the occurrence of other opportunistic infections during follow up were a day light fact [4]. But In sub-Saharan Africa, ART service has started recently and it is hard to learn the most important factors that can determine survival of patients on ART due to the limited scope and coverage of information available. As a result, the guidelines used in developing countries are adopted from studies done in developed countries with no consideration to local or regional settings. But out come of HIV treatment varies according to demographic, geographic, economic and cultural settings. Accordingly, characterizing the mortality of patients on ART treatment with respect to the economic, social, geographic and access to treatment care and support is very timely to respond according to local needs [5].

Immunological and virological responses to ART are similar to responses in patients treated in high-income countries. Despite this, however, early mortality rates in sub-Saharan Africa are very high; between 8% and 26% of patients die in the first year of ART, with most deaths occurring in the first few months. Mortality rates are likely to depend not only on the care delivered by ART programmes, but more fundamentally on how advanced disease is at programme enrolment and the quality of preceding health-care [5], [6].

The clinical benefit of ART for AIDS patients, in terms of mortality reduction and improved quality of life, is well established but shows regional variations, with higher case fatality rates in poor countries and factors contributing to this high mortality are poorly understood. There are several predictors of mortality for patients on ART: viral load, CD4 count, total lymphocytes,body mass index (BMI), and adherence [7], [8].So, a better knowledge of prognostic factors would allow closer follow-up and more targeted interventions in high risk patients, thus reducing excess mortality [6], [7], [8]. Like wise this study will enrich the available mass of evidence about mortality of patients on ART treatment in Ethiopia. For this reason, this study aims to investigate the predictors of mortality among adult patients on ART treatment.

## Methods

### Study Design and Setting

A retrospective cohort study was conducted among HIV patients on ART using records between September 2006 to February 2011. The study was conducted in Aksum hospital, Aksum town located 1067 north of Addis Ababa. The hospital serves as a referral for an estimated population of 1.2 million. The Hospital starts ART service on September 2005. All adults who were on ART treatment in Aksum Hospital HIV/AIDS clinic between September 2006 and February 2011 were included in the study. The clinic has 925 Patients enrolled on ART.

### Study Population and Sampling Techniques

The estimates of sample size or power were obtained for the test of the effect of one covariate, x_1_ (binary or continuous), on time to failure adjusted for other predictors, x_2_,…,x_k_, in a proportional hazard model. The sample size was calculated using the stpower Cox in stata version 11.0 software for the determination of sample size for Cox proportional hazards model. Calculation was done based on the assumption that type I error of 5%, power of 80% and the exposure is the CD4 cell count because it was the most significant predictor of mortality among adults on ART. For this reason individuals who have CD4 count of <200 cells/ul were at higher risk of dying than those who have above it (HR = 5.4) which gives the maximum sample size among the significant predictors of mortality in most literatures and, the standard deviation (sd = 0.5) was calculated from the mortality proportion(default value) [20]. Additionally the prevalence of low CD4(,200) 2.6% and lost to follow up were 12%. Based on this the total sample size was calculated to be 520. Regarding the sampling technique, record of study participants have been filtered first from the database according to their entry time to the follow up, next patients have been filtered using age and eligibility criteria then we give a unique number for the remaining records and select each record for our study using systematic random sampling. The out come variable was time event(death) of patients on ART.

### Data Collection Technique and Data Quality Control

A standard checklist was used for recording information extracted from electronic and paper based database and patient cards. This form is developed using the standardized ART entry and follow up form employed by the ART clinic. The CD4 count laboratory results recorded before starting ART were used as a base line values. If there is no pre-treatment laboratory test, however, results obtained within one month of ART initiation were considered as baseline values. The checklist consists of variables that are recorded at ART initiation (baseline) such as Sex, Age, Marital status, Educational status,CD4 cell, Baseline weight (kg), Haemoglobin (g/dL), WHO clinical stage, TB history before initiation of ART, Original ART regimen, CPT initiation and presence of OIs. Four experienced ART nurses who were trained on comprehensive HIV care and involved in patient follow ups collected the data and data collection was supervised by trained supervisors. During the data collection process the checklist was checked for their completeness, consistency and accuracy by the principal investigator every day. The data were entered and cleaned using SPSS version 16 and finally exported to stata version 11.0 for analysis.

### Data Management and Analysis

Exploratory data analysis was carried out to check the levels of missing values, presence of influential outliers, multicollinearity, normality and proportionality of hazards over time. The missed values were less than 4% in almost all of the factors. The proportion of the outcome were reported as alive, dead, lost to follow up and transferred out. The median follow up time of the cohort was also computed at 12,24 and 60 months. Person-months/years of follow up were calculated by assessing the date of enrolment for ART and death or censoring. The impact of the variables on patient survival was analysed using Kaplan-Meier survival analysis method. Log Rank test was used to test the equality of survival probabilities and compared across the different groups of covariates. The overall survival function and separate estimates for the stratum of covariates were considered as statistically significant at p-value <0.05 in the Log- rank test. Hazard ratios (HR) with 95% confidence intervals were used as effect measures. Multivariable Cox proportional hazards regression was used to model the contribution of baseline demographic characteristics and clinical characteristics on the mortality of adult persons on ART for variables with p<0.05 in bivariate analysis. This method has been used for the estimation of adjusted hazard ratios at 95% confidence intervals for the mortality rate among patients on ART for the covariates at their ART initiation. Model adequacy was assessed using Schoenfeld residuals.

**Baseline.** is the measurement taken nearest to the date of ART initiation.

### Ethical Statement

After assurance of anonymity, verbal consent for those who can’t read and write and written Consent for those who can read and write was obtained from all patients attending ART to store and use the information obtained during their first visit and subsequent follow ups. All information collected from patients cards were kept strictly confidential and names of patients on ART were not included in the data abstraction form. All the documentations and strategies used to maintain the Ethical principles has been provided to the Health research Ethics review committee of Mekelle University College of Health Science (HRERC) and clearance was secured after evaluation of the study protocol. Finally written support letter was obtained from Mekelle University and Tigray Regional Health Bureau to Aksum hospital. All patient data was accessed after the purpose of the research and ethical principles (confidentiality and security of database) had discussed with the hospital’s authority.

## Results

### Cohort Characteristics

A total of 520 patient records enrolled on ART were included in this study. The mean age and standard deviation of patients was (35.4±8.4) years. More than half (57%) of patients had no or primary education. Majority (60%) of patients were married **(** **Table 1** **)**. Most (72.8%) of patients were alive at the end of study period, 9.8% were lost to follow up, 8.5% had transferred out to other health facilities and 8.9% patients were died. The median follow up time for the cohort was 32 months with interquartile range of (22, 45)months. Around 78% of the participants have CD4 cell count of less than 200 cells/µl at baseline. Majority (78%) of the patients were under WHO stage III or IV. Around 43% of patients were having lower haemoglobin (anaemia) at baseline. The mean level of haemoglobin with standard deviation was (12.8±1.99) mg/dl. Thirteen percept of the patients have smeared positive pulmonary TB at baseline **(** **Table 2** **)**.

**Table 1:** Base line patients’ characteristics, 2006–2011, Aksum, Northern Ethiopia.

Variables	Number(520)	Event	Per cent
**Age**			
15–24 years	35	1	6.73
25–34 years	210	16	40.38
35–44 years	185	20	35.58
> = 45	90	9	17.31
**Gender**			
Male	225	24	43.27
Female	295	22	56.73
**Marital status**			
Never Married	101	15	19.42
Married	314	27	60.38
Divorced/Separated	55	3	10.58
Widowed	50	1	9.62
**Level of Education**			
No education	117	14	22.50
Primary	179	21	34.42
Secondary	180	11	34.62
Tertiary	44		8.46

**Table 2:** Base line Patients’ Clinical characteristics, 2006–2011, Aksum, Northern Ethiopia.

Variables	Number(N = 520)	Event	Per cent
**TB smear**			
Positive	71	11	13.65
Negative	346	31	66.54
Undetermined	103	4	19.81
**CD4 at baseline**			
<50	96	17	18.46
50–100	101	9	19.42
101–200	207	16	39.81
> = 201	116	4	22.31
**On Cotrimoxazol**			
Yes	429	38	82.50
No	91	8	17.50
**Weight at baseline**			
<40 kilograms	97	16	18.65
40–50 Kilograms	225	18	43.27
50–60 kilograms	147	10	28.27
>60 kilograms	51	2	9.81
**Functional status**			
Working	265	18	50.96
Ambulatory	214	21	41.15
Bedridden	41	7	7.88
**WHO stage**			
Stage I	33	2	6.35
Stage II	83	5	15.96
Stage III	336	24	64.62
Stage IV	68	15	13.08
**Haemoglobin at baseline**			
<11	223	17	42.88
> = 11	297	29	57.12

### Mortality and its Predictors

**Figure 1:**
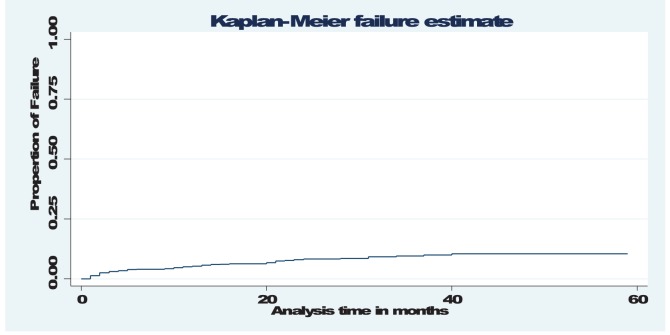
Kaplan - Meier probability of survival curve, 2006–2011, Aksum, Northern Ethiopia.

A total of 520 patients contributed to 1, 400 person-years of follow up and 46 patients died giving a mortality rate of 3.2 per 100 person- years. Of the 46 deaths, 27(59%) occurred within the first year of ART initiation which account a rate of 5.5 per 100 person- years (**Table 3** **and** **Figure 1**). On the other hand, the median survival time to event (dead) cases was 10 months with interquartile range of 2 to 21 months. However, the median time of stay on treatment was 14 months with interquartile range of 8 to 27 months.

**Table 3:** Survival of patients,September 2006 to August 2011, Aksum, Northern Ethiopia.

Months of follow up	Cumulative survival	95% confidence interval
12	0.94	0.923–0.963
24	0.91	0.889–0.938
36	0.90	0.872–0.928
48	0.89	0.860–0.921
60	0.89	0.860–0.921

In multivariate cox regression, patients with a CD4 cell count <50/µl had a 2-fold higher risk of mortality (Hazard ratio (HR) = 2.1, 95% CI: 1.12, 3.89) than individuals with CD4 count of >200/ul. Being male have also a 2 fold higher risk of mortality (HR = 1.9, 95% CI: 1.01, 3.52). Patients who have anaemia at baseline have almost 2 times higher risk of mortality than those patients who doesn’t have anaemia (HR = 1.9,95% CI: 1.01, 3.52). Like wise, patients with lower weight (<40 kg) have 2 fold higher risk of mortality than their counter parts(HR 2.4,95% CI: 1.24, 4.55) and those with lower education(no or primary education ) have 3 times increased risk of mortality than those individual who completed secondary or higher (HR = 2.61, 95% CI :1.17,5.80) and (HR = 2.67, 95% CI :1.28 − 5.48) respectively **(** **Table 4** **)**.

**Table 4:** Multivariate cox- regression of baseline predictors, 2006–2011, Aksum, Northern Ethiopia.

Variable	Adjusted Hazard ratio	95% confidence interval	P-value
**Age**			
15–24	1.00		
25–34	2.21	0.28,17.16	0.445
35–44	2.96	0.38, 23.01	0.298
≥45	2.63	0.30,22.62	0.376
**Sex**			
Male	1.88	1.00,3.52	0.047
Female	1.00		
**Marital status**			
Widowed	1.00		
Never married	2.04	0.99,3.82	0.06
Married	3.920	0.50,30.28	0.19
**Level of education**			
No education	2.60	1.17,5.80	0.019
Primary	2.67	1.28,5.54	0.008
Secondary and above	1.00		
**Weight at baseline (kg)**			
<40 kg	2.37	1.24,4.55	0.009
40–50	2.25	0.48, 10.47	0.299
51–60	1.88	0.39,8.97	0.428
≥60	1.00		
**CD4 count (cells/mm^3^)**			
<50	2.09	1.12,3.89	0.019
50–99	2.39	0.71,7.97	0.156
100–199	2.28	0.74,6.99	0.149
≥200	1.00		
**Haemoglobin**			
<11	2.25	1.23,4.14	0.008
> = 11	1.00		
**TB smear**			
Unknown	1.00		
Positive	2.62	0.91,7.50	0.07
Negative	1.33	0.65,2.71	0.43

Statistical Significance was declared at p-value <0.05.

## Discussion

In this study, mortality of patients enrolled on ART and factors that predict mortality of patients on ART was assessed. As a result, 8.85% died and majority (58.7%) of the deaths occurred in the first year of ART initiation and the highest 16(59%) mortality had happened in the first three months of the first year of ART initiation. In addition 34% of all deaths were within the first three months. Studies from Harar, Ethiopia, and rural hospitals of Tanzania and Malawi reported the same higher mortality occurring during the first three months of follow up, 49%, 62% and 61% respectively [8, 13, and 16].This is because most patients start ART at an advanced stage of the diseases. Another study from Senegal also shows higher mortality rate in the first year of ART initiation [9].

CD4 cell count <50/µl, haemoglobin level <11 mg/dl, weight < = 40 kg, gender, uneducated (illiterate) and primary level of education were found to be the strongest predictors of mortality among patients enrolled on ART.

Previous studies done in Africa shows that CD4 cell count less than 50 cell/µl was one of the major predictors of mortality for patients enrolled on ART [7], [9]–[12]. This study was consistent with those findings i.e. the risk of mortality was 2 times higher for those who have CD4 cell count less than 50 cell/µl compared to those who have CD4 count 200 cell/µl. marker of advanced immunodeficiency, was associated with opportunistic infection thereby increasing the likelihood ofdeath [21]. Patients who were anaemic at baseline were 2 fold higher risk of mortality than those who doesn’t have. The same study done across many parts of Africa reported that low level of haemoglobin at baseline is associated with high level of mortality among patients enrolled on ART [7–10, 15, 17, and 18].

Mortality rate was associated with baseline body weight of patients in this study. It was found that people who start ART with weight of <40 kg were dying at 2.37 times higher rate than those who have baseline body weight of >60 kg. In many other studies body mass index (BMI) was found to be the significant predictor of mortality but in the Ethiopian setting height of the patient was not recorded so that weight was used as proxy measure of BMI of patients. One studies done in Tanzania stated the same result that patients with lower body weight have higher risk of mortality compared to those with higher body weights [12], [14].This is because a weight loss of >5% has considerable prognostic value, not only for underweight patients but also for patients with normal weight at ART initiation [22].Males have around two times greater risk of dying than females which implies that male patients who start ART are dying at a greater rate of 1.8 times than females. When weighted against studies done in different countries, they itemized that males who are on ART have greater risk of dying than females who are on ART [7, 11, and 19]. Earlier health seeking behavior of women might explain the discrepancies of death among men and women [23] Additionally, Patients who have lower educational levels (no or primary education) at baseline were found to be 2.6 times more likely to die than those with higher educational level. As a result, patients with higher level of education might have a better understanding about the importance of compliance to their drugs and afford for the type of foods they need in a better way than those with low level of education. Additionally, According to reports, low educational level among patients is a contributing factor to late presentation for ART [24].

This study might suffer from limitations the retrospective cohort nature of the study and it also limits our ability to gather data about factors that may influence the risk of mortality, like information about patient behaviours, family supports, cares given at family level,adherence to treatment, etc.

In conclusion findings of this study point out that there were 46 deaths giving a mortality of 3.2 per 100 person-years of follow up. Of these, 27(59%) happened within the first year of ART initiation making the mortality rate 5.5 per 100 person- years. The final Cox proportional hazard model fitted identified CD4 count <50/ug, weight <40 kg, lower educational status (no or primary education) and anaemia at base line and male gender were found to be the predictors of mortality. For this reason, early initiation of ART despite the CD4 count and method of HIV diagnosis, nutritional support and close monitoring of patients in the early periods of ART treatment initiation is very crucial to improve patient survival.
